# A Case of COVID-19 Pneumonia Leading to Acute Respiratory Distress Syndrome (ARDS) and Multi-organ Failure Requiring Extracorporeal Membrane Oxygenation (ECMO) for Six Months and an Associated Critical Limb Ischemia

**DOI:** 10.7759/cureus.57329

**Published:** 2024-03-31

**Authors:** Kulsoom Durrani, Faiza Butt, Syed Atif, Erum Azhar, Abdul Waheed

**Affiliations:** 1 College of Medicine, Penn State University, Hershey, USA; 2 Family Medicine Residency Program, WellSpan Good Samaritan Hospital, Lebanon, USA; 3 Obstetrics and Gynecology, Creighton University School of Medicine, Phoenix, USA; 4 Obstetrics and Gynecology, Dignity Health East Valley, Gilbert, USA; 5 Family and Community Medicine, Creighton University School of Medicine, Phoenix, USA; 6 Family Medicine, Dignity Health Medical Group, Gilbert, USA

**Keywords:** acute respiratory distress syndrome (ards), complication of treatment, limb ischemia, coronavirus disease (covid-19), vv ecmo

## Abstract

This article presents the case of a 57-year-old woman with a history of rheumatoid arthritis who developed severe coronavirus disease 2019 (COVID-19) pneumonia that progressed to acute respiratory distress syndrome (ARDS) and multi-system organ failure. Despite initial slow progression and multiple hospital readmissions, her condition rapidly deteriorated, leading to full respiratory failure requiring intubation and ventilation. She was transferred to a specialized center where she underwent extracorporeal membrane oxygenation (ECMO) and hemodialysis for acute renal failure. Unfortunately, she remained dependent on ECMO for an extended period of six months. Although she made a gradual recovery, the prolonged critical care treatment resulted in critical ischemia of multiple extremities, necessitating a below-knee amputation (BKA) of her left lower extremity and transmetatarsal amputations of her right hand. This case reports one of the longest ECMO treatments for COVID-19 and associated comorbidities in the literature. Clinicians could include a longer duration of treatment and potential associated disabilities in the informed consent.

## Introduction

Coronavirus disease 2019 (COVID-19) is a contagious illness caused by severe acute respiratory syndrome coronavirus 2 (SARS-CoV-2). After the first case of COVID-19 was identified in Wuhan, Hubei Province, China, in December 2019, the virus was able to rapidly disseminate worldwide, causing the World Health Organization to declare it a global pandemic in March 2020. The pandemic caused a massive disruption to the world economy, a race to develop a vaccine, numerous lockdowns, and the death of over a million individuals in the United States alone by 2023 [1,2].

SARS-CoV-2 primarily targets the respiratory system and causes increased vascular permeability leading to the development of pulmonary edema. Viral mechanisms of injury include the following: direct viral injury, microthrombi deposition, dysregulation of the renin-angiotensin-aldosterone system, and activation of the kallikrein-bradykinin pathway. Extrapulmonary targets of COVID-19 include the gastrointestinal tract as well as the hepatobiliary, cardiovascular, and renal systems. Symptoms can range in severity from no illness to severe illness [1]. The National Institutes of Health (NIH) has issued guidelines that classify COVID-19 into five distinct categories: asymptomatic, mild illness, moderate illness, severe illness, and critical illness [3]. Most symptomatic individuals present with a mixture of fever, cough, shortness of breath, sore throat, and anosmia. In severe cases, patients may develop septic shock, multiple-organ dysfunction, and acute respiratory distress syndrome (ARDS) [1]. ARDS is a noncardiogenic pulmonary edema and lung inflammation characterized by acute onset, bilateral pulmonary infiltrates, and arterial blood oxygen to fraction oxygen in inspired air ratio (PaO2/FiO2) of less than 300. Mortality of ARDS is positively correlated with disease severity and ranges from 25% for mild disease to 45% for severe disease [4].

Risk factors for severe COVID-19 include age over 60 years, smoking, and the presence of underlying health conditions such as cardiovascular disease, obesity, diabetes, cancer, and lung disease [1]. In severe COVID-19 cases, extracorporeal membrane oxygenation (ECMO) may be necessary to sustain life. ECMO creates a circuit that is connected either veno-venous (VV) or veno-arterial (VA) to provide respiratory or cardiorespiratory support, respectively. The ECMO machine allows for bypass of the heart and lungs when cardiac or pulmonary dysfunction is present by acting as a pump with an oxygenator. ECMO draws blood out of the body, oxygenates it, removes waste products, and returns the blood into the systemic circulation. While ECMO is a life-saving tool, it can have severe complications which include hemorrhage, gas embolism, thrombosis, thromboembolism, sepsis, limb ischemia, and renal failure [5].

This case reports ARDS in the setting of COVID-19 with one of the longest ECMO treatments for COVID-19 and the associated comorbidities in the literature. Clinicians could include longer duration of treatment and potential associated disabilities in the informed consent.

## Case presentation

A 57-year-old woman with a history of rheumatoid arthritis on methotrexate was initially hospitalized at a local community hospital in South Central PA for COVID-19 pneumonia, requiring a 10-day stay. During this time, she received both remdesivir and dexamethasone for five days before being discharged home. However, she returned to the same community hospital 16 days later due to worsening shortness of breath and was admitted to the regular floor. Her condition deteriorated, leading to her transfer to the medical ICU at that same hospital on the 15th day of admission. Despite ongoing treatment, her respiratory failure worsened, necessitating intubation six days after her ICU transfer. On the same day, she was transferred to a quaternary/tertiary care hospital in South Central PA for evaluation of ECMO.

Upon arrival at the second hospital, the patient exhibited persistent hypoxemia, and her X-ray revealed diffuse infiltrates characteristic of COVID-19 and superimposed ARDS. Additionally, her arterial blood gases indicated severe respiratory acidosis with some metabolic acidosis that was persistent despite intubation and max ventilation support. Given her critical condition, she was promptly cannulated for VV ECMO. The patient did not have any risk factors or pulmonary comorbidities that could complicate decannulation from ECMO. During her 34-day stay at the higher-level facility, she underwent a tracheostomy and percutaneous endoscopic gastrostomy (PEG). Additionally, she experienced episodes of bacteremia and pneumonia caused by *Serratia marcescens* infections on days 30 and 36 of her hospitalization at the second hospital.

After 146 days since her initial presentation, the patient required continuous renal replacement therapy (CRRT) due to acute renal failure/acute kidney injury in the context of sepsis. She remained in the critical care unit throughout her stay at the second facility without any improvement in her condition. During her hospitalization, she experienced complications such as acute renal failure/acute kidney injury, which necessitated CRRT. CRRT was later transitioned to hemodialysis. Fortunately, the patient's renal function eventually recovered, allowing for the discontinuation of hemodialysis. Her eGFR was 55 mL/min/1.73m^2^ off of renal replacement therapy, and although she continued to recover, she still remained in the chronic kidney disease stage 3 range at discharge. She was able to be successfully decannulated from ECMO, and CRRT was discontinued 153 days after presentation to the higher level of care facility.

**Figure 1:**
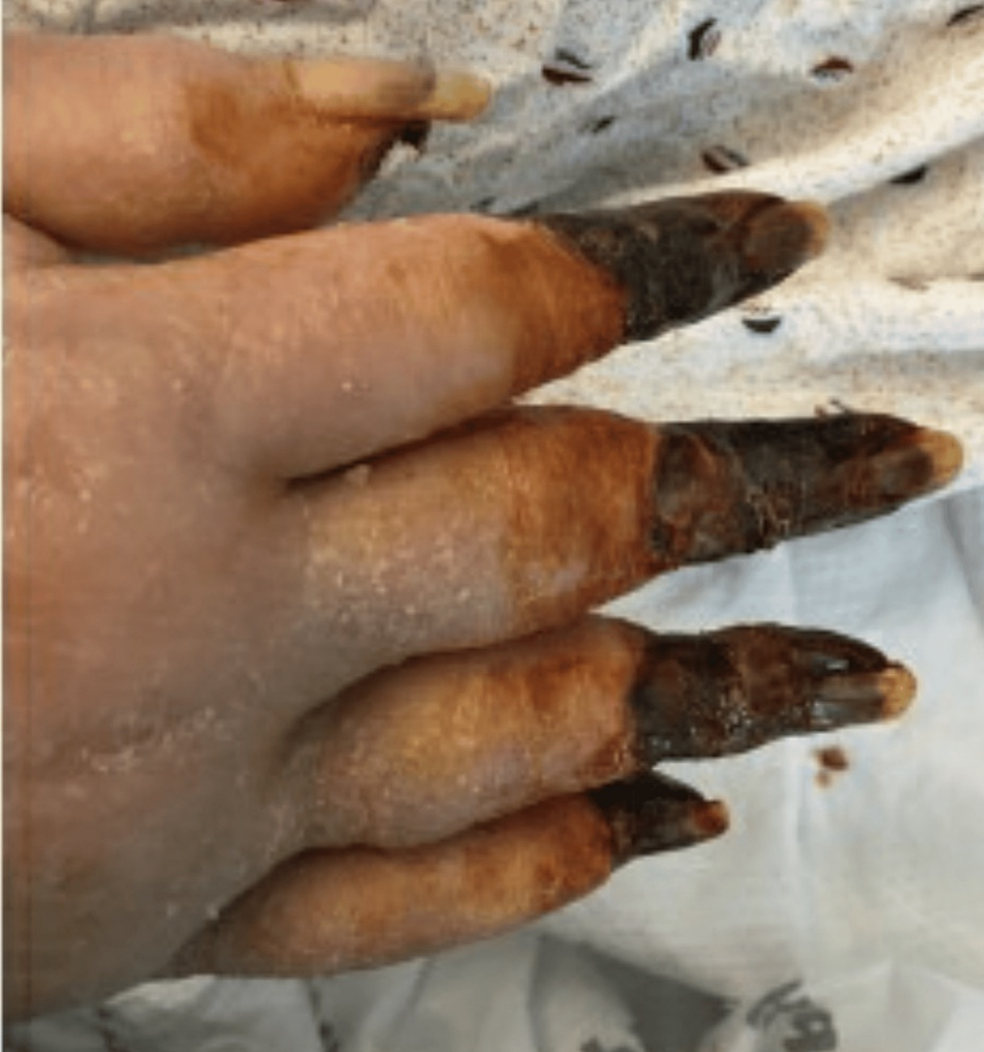
Right hand with gangrene affecting all digits

**Figure 2:**
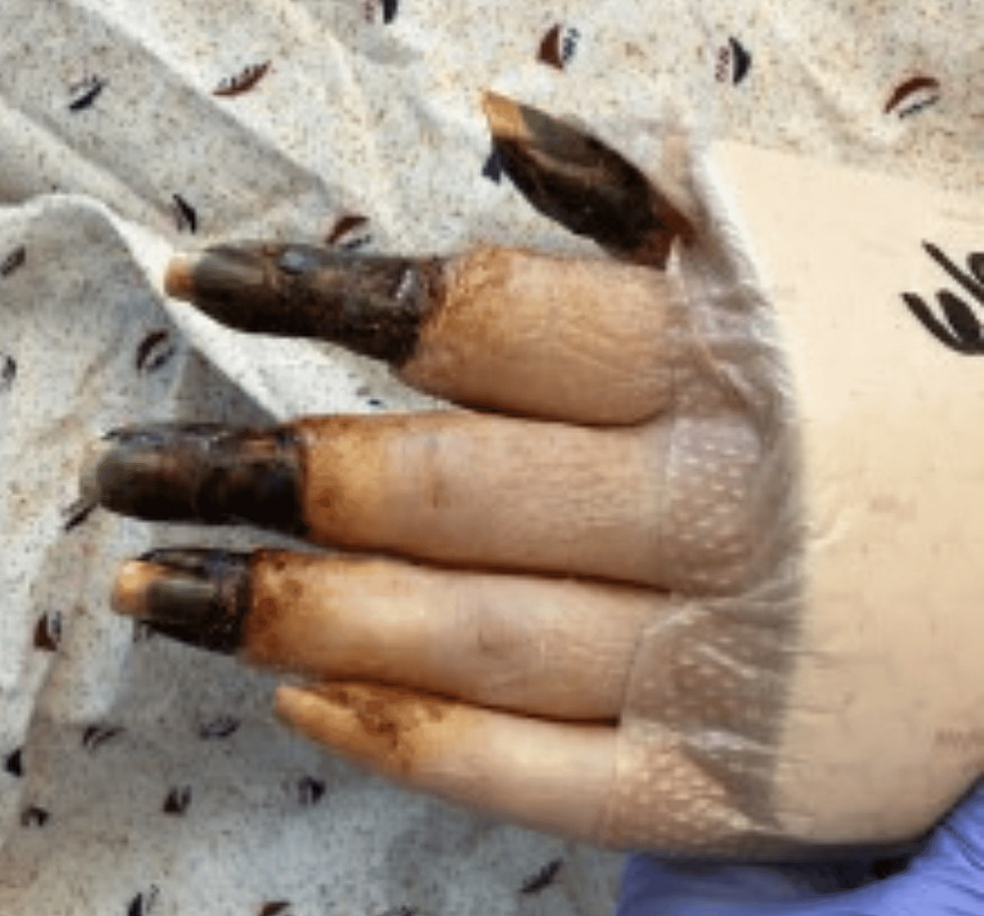
Left hand with gangrene affecting digits 1-3 and the thumb

**Figure 3:**
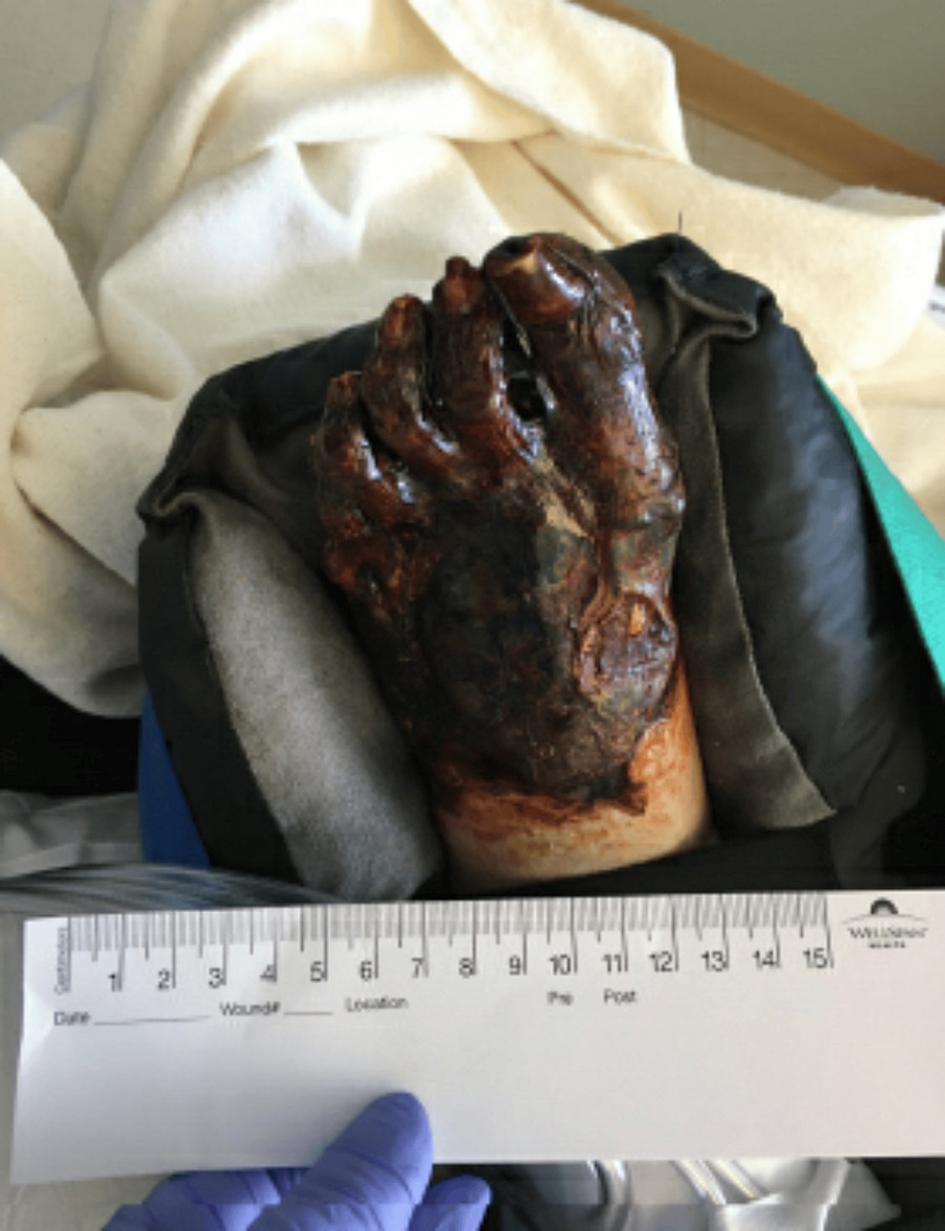
Left foot with gangrene affecting all digits up to the metatarsals

**Figure 4:**
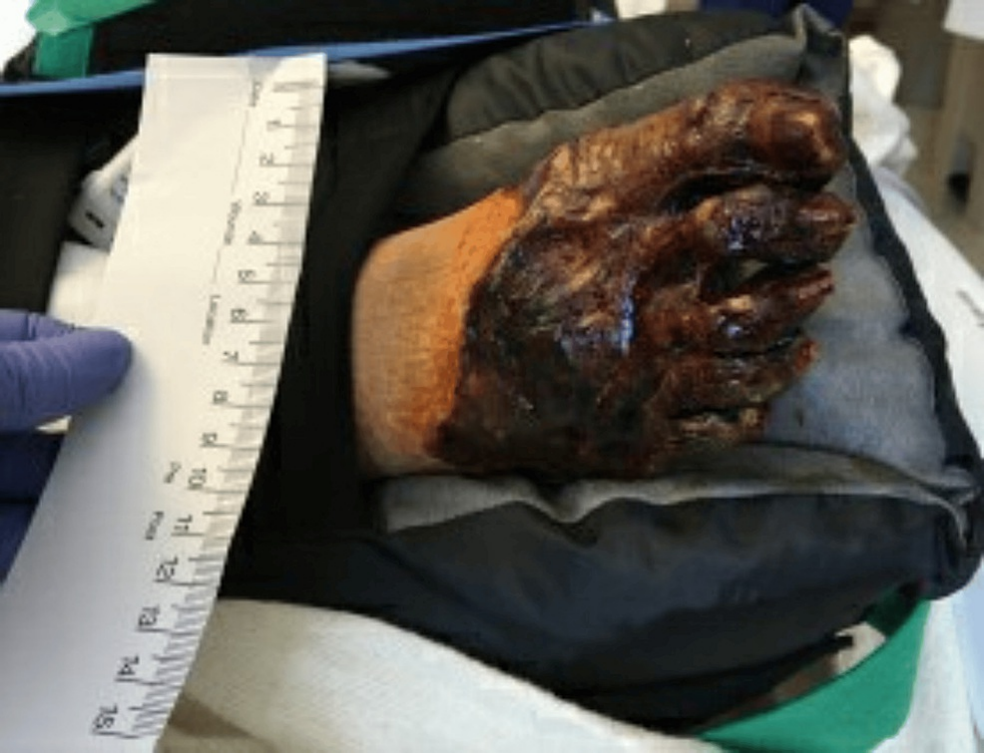
Right foot with gangrene affecting all digits up to the metatarsals

After ECMO decannulation, the patient remained stable on mechanical ventilation. However, necrosis of the distal aspects of all four extremities had set in by that time as shown in Figures 1-4.

Following her stabilization, the patient was transferred to a long-term acute care (LTAC) facility as she was not yet suitable for safe discharge home but did not require more intensive medical interventions. During her stay at the LTAC, she was gradually weaned off vasopressors and sedation, became able to tolerate pressure support trials, and was safely extubated. Her tracheostomy was also decannulated. Subsequently, she was transferred from the LTAC to the University of Pittsburgh Medical Center (UPMC) for a left below-knee amputation (BKA) due to the development of dry gangrene as a complication of COVID-19 and prolonged ECMO support. The left BKA was performed 190 days after her initial presentation without any complications, and her pain was well-managed. After the successful procedure, the patient was returned to the LTAC facility. She was reassessed by orthopedic surgery 23 days after the left BKA, and prophylactic Bactrim was initiated. Later on, she underwent a right transmetatarsal amputation of the hand in zones 1C and 1D.

Prior to the second amputation, she was hemodynamically stable and was discharged to an acute inpatient rehab and followed up with the surgery team as an outpatient. The patient was admitted to the inpatient rehab of the community for a comprehensive inpatient rehabilitation program consisting of physical therapy, occupational therapy, speech and language pathology, psychology, rehabilitation nursing, and case management support. Because the patient still had proximal extremity muscle weakness from her prolonged ICU and ECMO course, she was discharged from the rehabilitation program with a power mobility device, physical therapy, and occupational therapy to compensate for her difficulties with activities of daily weakness as well as to rebuild her muscle strength.

Additionally, the patient received two doses of Pfizer-BioNTech COVID-19 vaccine during her hospitalization, after decannulation from the ECMO.

## Discussion

Since the start of the COVID-19 pandemic, many treatments have been used to treat COVID-19 with varying levels of success. ECMO is a life-saving measure that allows for the preservation of lung function in the setting of lung injury due to severe respiratory distress syndrome. ECMO has even been incorporated as part of the standard treatment algorithm in many health systems for patients with severe refractory ARDS. Despite the potential benefits of ECMO, it is not benign, and side effects scale with the duration of treatment. Side effects of ECMO include venous thromboembolism, coagulopathy, infection, limb ischemia, seizures, cerebrovascular accident, and others [5].

The Extracorporeal Life Support Organization (ELSO) outlines indications for VV ECMO that include hypoxic respiratory failure when the predicted risk of mortality is 50% or greater (e.g., determined by Sequential Organ Failure Assessment, SOFA; Simplified Acute Physiology Score 2, SAPS-2; Acute Physiologic Score and Chronic Health Evaluation, APACHE score; etc.), hypercapnic respiratory failure (blood pH less than 7.25) despite mechanical ventilation with high inspiratory plateau pressure (>30 cm H2O), duration of time on ventilator is less than 10 days, and age less than 65 years [6].

Relative contraindications for VV ECMO include a BMI greater than or equal to 40 and an age greater than or equal to 65. Absolute contraindications are advanced age, multi-organ failure, advanced lung disease, uncontrolled diabetes mellitus, acute neurologic injury, and severe peripheral vascular disease [7].

Whether or not this patient met the existing criteria for VV ECMO is a critical learning and discussion point for this case. According to a review of the patient’s record, she was less than 65 years old and in severe hypoxemic respiratory failure with hypercarbia and acidosis. She was also intubated and cannulated for ECMO within 10 days of beginning mechanical ventilator treatment, and she was without the contraindications mentioned previously. Consequently, she qualified for VV ECMO.

Our second critical learning and discussion point was if anything could have been done differently, considering that the patient experienced multiple limb ischemia likely secondary to her prolonged ECMO treatment, as limb ischemia is a well-documented side effect of prolonged ECMO. While hindsight is 20/20, we must remember that this case occurred at the beginning of the COVID-19 pandemic, and little was known about the virus at that time. Theoretically, ECMO could have been performed prior to intubation, the so-called "awake ECMO" that was successfully performed by Azzam et al. This could have allowed for the mitigation of ventilator-associated barotrauma. However, as Azzam et al. have highlighted, ECMO initiation prior to intubation is an experimental management approach [8]. Consequently, it is difficult to tell if initiating ECMO prior to intubation would have resulted in a significantly different clinical course for this patient.

Unfortunately, this patient was infected with SARS-CoV-2 a month prior to when she would have been eligible to receive the first COVID-19 vaccine as a healthcare worker. If the patient had received the vaccine prior to her illness, it is possible that she would have had a less severe disease course. Throughout the duration of the pandemic, even after the availability of COVID-19 vaccines, multiple treatment options have been explored to treat and prevent severe COVID-19, with varying levels of efficacy, including hydroxychloroquine, convalescent plasma, remdesivir, dexamethasone, numerous monoclonal antibodies, and even controversial treatments including ivermectin. In this case, the patient’s treatment seemed appropriate at the time and likely saved her life. During this patient’s hospital course, the scientific community continued to learn about the SARS-CoV-2 virus, and there was no standardized treatment protocol to manage COVID-19. Further complicating matters, the SARS-CoV-2 virus continued to and still continues to evolve new variants, making the development of standard treatment algorithms difficult. Consequently, it will likely be many years before COVID-19 becomes a predictable disease with definitive and standardized treatments.

## Conclusions

This case reports ARDS in the setting of COVID-19 with unusually long ECMO treatment for COVID-19 pneumonia and associated comorbidities. Clinicians should include longer duration of treatment and resulting disabilities in the informed consent for ECMO to enhance informed consent and shared decision making. While ECMO treatment can have tremendous lifesaving benefits, there are significant potential consequences including comorbidities and cost. 

Early in the COVID-19 pandemic, the virus was still unknown, and there were no clear guidelines on treatment or vaccines. This rapidly evolving situation led to difficult decision-making, especially as the virus continued to mutate into new strains. This case is an opportunity for clinicians and patients to learn to weigh the risks and the benefits of treatment options in ambiguous cases.
